# Differential Epidemiology of *Salmonella* Typhi and Paratyphi A in Kathmandu, Nepal: A Matched Case Control Investigation in a Highly Endemic Enteric Fever Setting

**DOI:** 10.1371/journal.pntd.0002391

**Published:** 2013-08-22

**Authors:** Abhilasha Karkey, Corinne N. Thompson, Nga Tran Vu Thieu, Sabina Dongol, Tu Le Thi Phuong, Phat Voong Vinh, Amit Arjyal, Laura B. Martin, Simona Rondini, Jeremy J. Farrar, Christiane Dolecek, Buddha Basnyat, Stephen Baker

**Affiliations:** 1 Oxford University Clinical Research Unit, Patan Academy of Health Sciences, Kathmandu, Nepal; 2 The Hospital for Tropical Diseases, Wellcome Trust Major Overseas Programme, Oxford University Clinical Research Unit, Ho Chi Minh City, Vietnam; 3 Centre for Tropical Medicine, Oxford University, Oxford, United Kingdom; 4 Novartis Institute Vaccines for Global Health, Siena, Italy; 5 The London School of Hygiene and Tropical Medicine, London, United Kingdom; University of Otago, New Zealand

## Abstract

**Background:**

Enteric fever, a systemic infection caused by the bacteria *Salmonella* Typhi and *Salmonella* Paratyphi A, is endemic in Kathmandu, Nepal. Previous work identified proximity to poor quality water sources as a community-level risk for infection. Here, we sought to examine individual-level risk factors related to hygiene and sanitation to improve our understanding of the epidemiology of enteric fever in this setting.

**Methodology and principal findings:**

A matched case-control analysis was performed through enrollment of 103 blood culture positive enteric fever patients and 294 afebrile community-based age and gender-matched controls. A detailed questionnaire was administered to both cases and controls and the association between enteric fever infection and potential exposures were examined through conditional logistic regression. Several behavioral practices were identified as protective against infection with enteric fever, including water storage and hygienic habits. Additionally, we found that exposures related to poor water and socioeconomic status are more influential in the risk of infection with *S.* Typhi, whereas food consumption habits and migration play more of a role in risk of *S.* Paratyphi A infection.

**Conclusions and significance:**

Our work suggests that *S.* Typhi and *S.* Paratyphi A follow different routes of infection in this highly endemic setting and that sustained exposure to both serovars probably leads to the development of passive immunity. In the absence of a polyvalent vaccine against *S.* Typhi and *S.* Paratyphi A, we advocate better systems for water treatment and storage, improvements in the quality of street food, and vaccination with currently available *S.* Typhi vaccines.

## Author Summary

Enteric fever, caused by ingestion of bacteria *Salmonella* Typhi or *Salmonella* Paratyphi A, is common in regions with poor water quality and sanitation. We sought to identify individual-level risks for infection in Kathmandu, Nepal, a region endemic for enteric fever. In this study, we enrolled patients presenting to hospital who were blood-culture positive for enteric fever and a series of community controls matched for age, gender and residential ward. Our findings suggest that while some risks for infection with *S.* Typhi and *S.* Paratyphi A overlap, these organisms also have distinctive routes of infection in this setting; poor water and socioeconomic status seemed more influential in infection with *S.* Typhi, whereas food consumption habits and migratory status were shown to play a larger role in infection with *S.* Paratyphi A. Additionally, serological evaluation of IgG levels against the Vi (*Salmonella* Typhi) and the O:2 (*Salmonella* Paratyphi A) antigens demonstrated high titers against both antigens throughout life, suggesting frequent and constant exposure to these organisms in Kathmandu. As major improvements in sanitation infrastructure are unlikely in this setting, we recommend water treatment and storage-based prevention strategies, as well as street food quality regulation, and the promotion of vaccination with existing typhoid vaccines.

## Introduction

The human systemic disease enteric fever is most commonly caused by the *Salmonella enterica* serovars Typhi (*S.* Typhi) and Paratyphi A (*S.* Paratyphi A) [1], [2]. The disease is found in areas with poor sanitation and hygiene [3], and has an estimated global burden of 27 million new cases and 200,000 deaths annually [4]. The causative bacteria are transmitted fecal-orally. After ingestion, a 7 to 14 day symptomatic period ensues whereupon bacteremia presents as a persistent non-focal fever with malaise. The bacteria can induce a protracted illness that lasts several weeks, and while rarely fatal, the disease can result in life threatening complications including hypotensive shock and intestinal perforation [2], [5].

Enteric fever is endemic in Nepal and *S.* Typhi and *S.* Paratyphi A are the most commonly isolated organisms from the blood of febrile patients in our Kathmandu-based healthcare setting [6], [7]. A retrospective analysis highlighted a substantial burden of enteric fever within the local population, particularly in school-age children and males aged 15 to 25 years [8]. Furthermore, we have shown that indirect transmission through contaminated drinking water may play a more important role in maintaining the endemicity of infection in Kathmandu than close contact with symptomatic or asymptomatic individuals [9], [10]. However, there are several gaps in our knowledge of the transmission of enteric fever and specific behavioral risk factors for infection in Kathmandu have not been identified.

Several case-control studies have investigated risks for enteric fever; the majority implicate water and food as important transmission routes [11]–[21]. Additional risk factors include previous contact with an enteric fever case [12], [19], [22], recent antimicrobial treatment [15], local flooding [19], poor hygiene [13], [14] and poor socioeconomic status [13], [19]. The identification of tractable risk factors and probable routes of transmission for the agents of enteric fever are necessary for the development of targeted interventions to reduce disease burden. In this study, a case-control investigation and age-stratified serology were performed to identify risk factors for, and measure expose to, enteric fever in Kathmandu.

## Methods

### Ethical approval

This study was approved by the institutional ethical review boards of Patan Hospital and The Nepal Health Research Council. All enrollees were required to provide written informed consent for the collection and storage of all samples and subsequent data analysis. In the case of those under 18 years of age, a parent or guardian was asked to provide written informed consent.

### Study site

**Figure 1:**
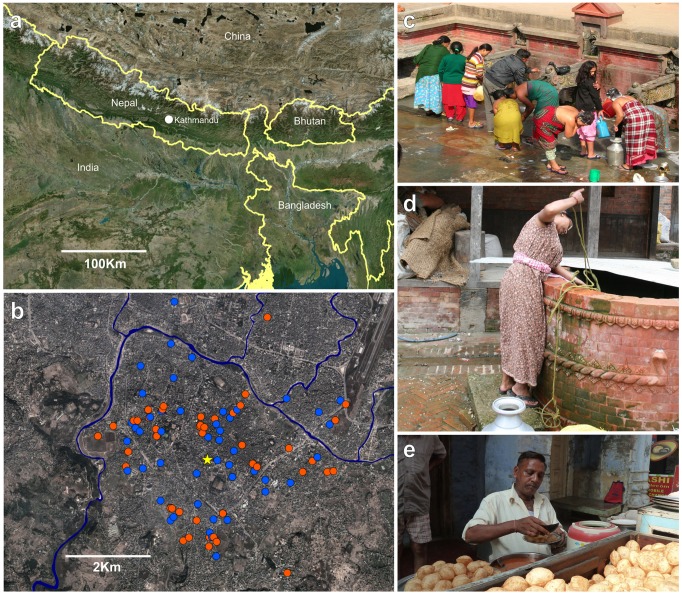
The location and the socioeconomics of Kathmandu. a) Map of southern Asia showing the location of Nepal and the capital city of Kathmandu; the location for the case/control investigation, in the context of neighboring countries b) Lalitpur, Kathmandu; the locale of the case control study, the locations of household of the cases are shown in red (*S.* Typhi) and blue (*S.* Paratyphi A), and the site of enrollment of the cases (Patan hospital) is shown by a yellow star. c) A typical municipal stone waterspout in the location of the enteric fever case/control investigation. d) A typical municipal sunken well in the location of the enteric fever case/control investigation. e) A local street food vendor preparing and selling pani puri.

Patan Hospital is a 318-bed government hospital providing emergency and elective outpatient and inpatient services located in Lalitpur Sub-metropolitan City (LSMC) within the Kathmandu Valley (Figure 1). Enteric fever is common at the outpatient clinic at Patan Hospital, which has approximately 200,000 outpatient visits annually. The population of LSMC is generally poor, with most living in overcrowded conditions and obtaining their water from stone spouts or sunken wells (Figure 1).

### Enrollment and questionnaire

All febrile patients attending the outpatient or emergency department between April and October 2011 with a non-focal fever lasting 3 or more days, aged between 2–65 years and providing informed consent were eligible for this study. All individuals received a blood culture and only those with a blood culture positive for *S.* Typhi or *S.* Paratyphi A were enrolled.

Community-based controls were matched for age, sex and residential ward and enrolled at a ratio of 3∶1. Controls were identified in households neighboring cases. If the case lived in a stand-alone house, the household to the right of the “case-household” was approached by a community medical assistant (CMA) within 2 weeks of the case enrollment. If this control refused, the household to the left was approached, followed by the house parallel across the street. If the case lived in multi-story building, the household above the “case-household” was approached. If this control refused, a household a storey either below or two stories below was approached. Controls were required to be within 5 years of the age of the case and must not have had fever, gastrointestinal disturbances or history of enteric fever in the month before administration of the questionnaire. If an approached control failed to meet the enrollment criteria or refused participation, the house to the left or the storey below the “case-household” was approached for enrollment. The trained CMAs administered a 129-question questionnaire to each enrolled individual.

### Blood culture

Anti-coagulated blood samples were collected from all febrile patients upon arrival in the outpatient department. For those over the age of 12 years, 10 ml of blood sample was collected; 5 ml was collected from those aged 12 years or less. The blood samples were inoculated into tryptone soya broth and sodium polyethanol sulphonate up to 50 ml. The inoculated media was incubated at 37°C and examined daily for bacterial growth over seven days. On observation of turbidity, the media was sub-cultured onto MacConkey agar. Any bacterial growth presumptive of *S.* Typhi or *S.* Paratyphi was identified using serotype specific antisera (Murex Biotech, Dartford, UK).

### Serological testing

ELISAs to measure IgG against the Vi and O:2 antigens (NVGH, Siena, Italy) [23], [24] were performed on 795 plasma samples that were age-stratified and randomly selected from a serum bank comprised from the blood of patients attending Emergency Department of the Patan Hospital for reasons other than typhoid treatment or those relating to a febrile illness between January 2009–December 2011. Ages of enrollees ranged from 0–65 and were resident in the same demographic area as the case/control enrollees. All plasma samples were subjected to ELISA detecting IgG antibodies to both Vi and O:2. Briefly, plasma was diluted into 1∶200 and aliquoted into ELISA plates (Nunc, Sigma-Aldrich Co, UK) independently coated with Vi and O:2 antigens. After washing, bound IgG was detected using an alkaline phosphatase–conjugate antiserum (Sigma-Aldrich Co, UK). Antibody levels were quantified using standard curves. The standard curve for Vi-antibody was created using a prepared anti-human Vi-antibody standard. The standard curve for O:2-antibody was created using a pool of plasma from *S.* Paratyphi A confirmed patients who had high levels of antibody to O:2, which had been screened prior to the serum bank. The cutoff value of the ELISAs was defined as the optical density of blank control wells plus two standard deviations.

### Statistical methods

Data were imported into STATA v9.2 (College Station, TX, USA). The association between the outcome of enteric fever (defined as infection with either *S.* Typhi or *S.* Paratyphi A) and each exposure of interest was examined using a matched univariate analysis through conditional logistic regression. A conceptual model was generated to develop a biologically plausible set of covariates *a-priori* that were thought likely to influence the outcome, including those related to poor socioeconomic status and poor water quality. Variables were included in a matched multivariate analysis through *a-priori* selection or were associated (p<0.25) with enteric fever in the univariate analysis [25]. Model fit was assessed through log likelihood and relative AIC value. Co-linearity among variables was assessed but no strong associations were found between the variables included in the final model. The presence of effect modification was evaluated between each of the salient exposures and confounders of interest through the χ^2^ test for homogeneity. Several sets of interactions were found to be significant. Each interaction term was included in the final model of interest and model fit was assessed. However, due to small numbers of patients in many of the strata, inclusions of these interactions led to an unstable estimate and were thus discarded. One interaction, however, household size affecting the risk of water storage on outcome of enteric fever, improved model fit and led to stable estimates so was included. The categorical variable household size was generated through assessing whether the house was the same size or smaller than the median number of people (12) in this dataset or larger than the median. All *P* values are two-sided.

## Results

### Baseline and clinical characteristics

During the period of investigation 103 febrile patients with culture confirmed enteric fever were enrolled, 48% (49/103) were positive for *S.* Typhi and 52% (54/103) were positive for *S.* Paratyphi A. Baseline characteristics of the enteric fever cases are described in Table 1. Briefly, those with culture confirmed enteric fever were more often male (64%; 66/103) and had a median age of 18 years (interquartile range (IQR) 10–23 years). Males and females with *S.* Paratyphi A did not differ significantly in age (median: 18 years, IQR: 5–55 and 20 years, IQR: 6–28, respectively), but female *S.* Typhi cases were significantly older (median: 21.5 years, IQR: 8–50) than male *S.* Typhi cases (median: 16 years, IQR: 7–32) (*P* = 0.03, Mann-Whitney U test). A total of 294 controls were enrolled, leading to a final case-control ratio of 1∶2.85.

**Table 1:** Baseline characteristics from *S.* Paratyphi A and *S.* Typhi cases and matched controls in Kathmandu, Nepal, 2011.

Variable	*S.* Paratyphi A		*S.* Typhi		Total	
	Cases	Controls	Cases	Controls	Cases	Controls
	n = 54 (%)	n = 158 (%)	n = 49 (%)	n = 136 (%)	n = 103 (%)	n = 294 (%)
**Demographic**						
Age (mean (range))	17.6 (5–55)	17.3 (1–51)	18.6 (7–50)^a^	17.4 (2–38)	18 (5–55)	17 (0–53)
Male sex	37 (68.5)	110 (69.6)	29 (59.2)	83 (61.0)	66 (64.1)	193 (65.7)
**General enteric fever**						
Aware of enteric fever	16 (29.6)	91 (57.6)	13 (26.5)	68 (50.0)	29 (28.2)	159 (54.1)
Previous episode of enteric fever	10 (18.5)	45 (28.5)	8 (16.3)	37 (27.2)	18 (17.5)	82 (27.9)
Enteric fever contact <8 weeks	7 (13.0)	58 (36.7)	4 (8.2)	57 (41.9)	11 (10.7)	1115 (39.1)
Vi vaccination	1 (1.9)	1 (0.6)	2 (4.1)	10 (7.4)	11 (3.7)	3 (2.9)
**Socioeconomic status**						
Household income <$125/month	19 (35.2)	31 (20.0)^b^	16 (32.7)	27 (19.9)	59 (57.3)	197 (67.7)
Owner of a motorbike	13 (24.1)	37 (23.4)	8 (16.3)	44 (32.4)	21 (20.4)	81 (27.6)
Household size (median (IQR))^h^	13 (8–20)	12 (5–22)	13 (7–25)	12 (5–25)	13 (8–21)	12 (5–23)
Duration of stay in KTM <2 years	23 (46.9)^c^	47 (31.3)^c^	17 (37.8)^f^	40 (32.0)^g^	40 (42.6)	87 (31.6)
**Water supply**						
Municipal (piped)	29 (53.7)	119 (75.3)	26 (53.1)	92 (67.6)	64 (62.1)	190 (64.6)
Stone spout	12 (24.1)	30 (19.0)	27 (55.1)	56 (41.2)	38 (36.9)	85 (28.9)
Well	36 (66.7)	109 (69.0)	19 (38.8)	79 (58.1)	68 (66.0)	218 (74.2)
Water shortage affects daily life	29 (53.7)	119 (75.3)	30 (61.2)	106 (77.9)	59 (57.3)	225 (76.5)
**Local Conditions**						
Household or neighborhood floods	13 (24.1)	30 (19.0)	6 (12.5)	19 (14.0)	19 (18.6)	49 (16.7)
Garbage or defecation visible	25 (46.3)	82 (51.9)	31 (63.3)	86 (63.2)	56 (54.4)	168 (57.1)
**Water storage**						
Water treated	43 (79.6)	121 (75.8)^d^	33 (67.3)	95 (69.9)	76 (73.8)	214 (73.0)
Water stored after collection	41 (78.9)	116 (77.3)	42 (87.5)^f^	94 (69.6)^e^	83 (83.0)	210 (73.7)
Wide mouth of storage container	9 (16.7)	46 (29.1)	6 (12.2)	36 (26.5)	15 (14.6)	82 (27.9)
Metal covering of water storage	7 (13.0)	59 (37.3)	7 (14.2)	54 (39.7)	14 (14.0)	113 (38.4)
**Sanitation**						
Hands washed after toilet	52 (96.3)	151 (96.2)	48 (98.0)	135 (99.3)	100 (97.1)	286 (97.6)
Hands wiped after washing	29 (53.7)	126 (81.8)^d^	25 (51.0)	96 (70.6)	54 (52.9)	222 (77.4)
**Latrine**						
Household latrine	49 (90.7)	118 (76.1)	46 (95.8)	108 (79.4)	95 (92.2)	226 (77.9)
Number using latrine (median(IQR))	8 (6–12)	9 (6–20)	9 (5–12)	10 (6–17)	8 (6–12)	10 (6–20)
**Food consumption**						
Dairy consumption	3 (7.0)^i^	10 (6.9)^i^	6 (19.4)^j^	12 (10.0)^j^	9 (12.2)	22 (8.3)
Pani puri consumption^k^	17 (31.5)	76 (48.1)	17 (34.7)	67 (49.3)	34 (48.6)	143 (58.6)
Eaten street food<2weeks	22 (40.7)	50 (31.6)	24 (49.0)	52 (38.2)	54 (52.4)	109 (37.1)

a : no controls were identified for one case aged 50 years; data from.

b : 155 controls;

c : 49 cases, 150 controls;

d : 157 controls;

e : 154 controls;

f : 45 cases; 125 controls;

g : 48 cases, 135 controls,

h : the number of people sharing a kitchen;

i : 43 cases, 146 controls;

j : 31 cases, 100 controls;

k : panipuri is a local bread snack that is dipped is a water-based sauce.

The clinical presentations of *S.* Typhi and *S.* Paratyphi A were largely indistinguishable, with most cases exhibiting a progressive fever (77%; 79/103), nausea (50%; 51/103), and a limited number having an abdominal rash (2%; 2/103) or constipation (6%; 6/103). Patients with *S.* Typhi were more likely to present with abdominal pain (61%; 30/49) than those with *S.* Paratyphi A (33%; 18/54) (*P* = 0.005, χ^2^ test) and to have diarrhea (25%; 12/49 and 9%; 5/54, respectively (*P* = 0.038, χ^2^ test)).

### General risks for enteric fever

To identify important associations between various exposures and the outcome of enteric fever due to either *S.* Typhi or *S.* Paratyphi A, we performed a series of univariate analyses and, to control for confounding, built a multivariate model. As shown in Table 2, a variety of exposures were found to be protective against enteric fever in a univariate analysis. These protective variables included, an awareness of enteric fever, reporting a previous enteric fever episode, recent contact with an enteric fever case, using a metal cover on a household water storage container, and the consumption of pani puri. The counterintuitive protective effects of recent contact with an enteric fever case and consumption of pani puri were considered to be a result of study design. As cases and controls were geographically matched, it is likely that controls were aware of local cases. Additionally, as pani puri has the potential to be fecally contaminated [26], the protective association is likely to be explained by uncollected information. These exposures were not included in the final model. Enteric fever risks from the univariate analysis included a household monthly income of <$125, the duration of residence in Kathmandu, the use of stone spout water, storing water in the household, the use of a household latrine, and the recent consumption of street food. From the multivariate model, an awareness of enteric fever (AOR: 0.25, 95%CI: 0.1–0.5, p<0.001) and the use of a metal cover on household water storage (AOR: 0.28, 95%CI: 0.1–0.7, p = 0.006) remained significantly protective against infection. The use of a household latrine, compared to a community latrine (AOR: 4.10, 95%CI: 1.4–12.4, p = 0.013), as well as recent street food consumption (AOR: 2.85, 95%CI: 1.4–6.0, p = 0.006) remained as strong risk factors for infection. Notably, the risk of storing water was found to vary by household size. Those living in households with more than 12 people were at significant risk of infection if they stored water (AOR: 9.35, 95%CI: 1.7–51.0, p = 0.010), while those who lived in smaller houses were unaffected by water storage habits.

**Table 2:** Matched and adjusted odds ratios for various exposures for enteric fever cases and matched controls in Kathmandu, Nepal, 2011.

Variable	MOR	95%CI	p	AOR	95%CI	p
Awareness of enteric fever	0.26	0.2–0.4	<0.001	0.25	0.1–0.5	<0.001
Previous episode of enteric fever	0.47	0.3–0.9	0.016	0.57	0.3–1.3	0.187
Enteric fever contact <8 weeks	0.12	0.1–0.3	<0.001			
Household size >12 people	1.60	0.9–2.8	0.102	0.24	0.1–1.1	0.065
Household monthly income <$125	2.65	1.5–4.7	0.001	1.87	0.9–4.1	0.118
Duration of stay in KTM <2 years	1.71	1.0–2.9	0.053	1.41	0.7–2.8	0.338
Local flooding	1.23	0.6–2.5	0.567			
Local unsanitary conditions^a^	0.82	0.5–1.4	0.481			
Use of stone spout water	2.32	1.1–5.0	0.033	0.96	0.3–3.0	0.940
Water stored after collection	1.93	1.0–3.6	0.043	0.68	0.2–2.3	0.534
Metal covering of water storage	0.17	0.1–0.4	<0.001	0.28	0.1–0.7	0.006
Latrine type						
Community	1.00	-				
Household	5.71	2.3–14.4	<0.001	4.10	1.4–12.4	0.013
Eaten street food<2weeks	2.34	1.4–4.0	0.002	2.85	1.4–6.0	0.006
Dairy consumption	1.29	0.4–3.7	0.645			
Pani puri consumption	0.42	0.2–0.8	0.013			
Water stored & household size >12^b^				9.35	1.7–51.0	0.010

MOR: matched odds ratio; AOR: adjusted matched odds ratio; 95%CI: 95% confidence interval; KTM: Kathmandu;

a : garbage or defecation visible in neighborhood;

b : significant interaction term, test for homogeneity of odds ratios p = 0.017.

### Specific risks for *S.* Paratyphi A infection

From the series of univariate analyses performed on *S.* Paratyphi A cases and matched controls only, many of the identified protective and risk factors, as shown in Table 3, were similar to the overall enteric fever analysis shown in Table 2. However, the final multivariate model demonstrated that the use of a metal cover for water storage (AOR: 0.19, 95%CI: 0.1–0.7, p = 0.014) remained strongly protective against infection with *S.* Paratyphi A, whereas the consumption of street food within the two weeks preceding illness remained a significant risk factor (AOR: 2.95; 95%CI: 1.1–7.8, p = 0.028). Furthermore, residing in Kathmandu for less than two years (AOR: 2.29, 95%CI: 0.9–5.7, p = 0.077) remained a weakly significant risk factor for *S.* Paratyphi A infection only.

**Table 3:** Matched and adjusted odds ratios for various exposures for *S.* Paratyphi A and *S.* Typhi cases and matched controls in Kathmandu, Nepal, 2011.

Variable	*S.* Paratyphi A						*S.* Typhi					
	MOR	95%CI	p	AOR	95%CI	p	MOR	95%CI	p	AOR	95%CI	p
Awareness of enteric fever	0.26	0.1–0.5	<0.001	0.60	0.3–1.3	0.180	0.26	0.1–0.6	0.001	0.28	0.1–0.8	0.014
Previous episode of enteric fever	0.57	0.3–1.3	0.166	0.83	0.3–2.1	0.700	0.34	0.1–0.9	0.040	0.41	0.1–1.5	0.182
Enteric fever contact <8 weeks	0.15	0.1–0.5	0.001				0.10	0.0–0.3	<0.001			
Household size >12 people	1.80	0.8–4.0	0.145				1.41	0.6–3.1	0.399	2.11	0.8–5.5	0.127
Household monthly income <$125	2.75	1.3–6.3	0.012	1.15	0.4–3.3	0.787	2.55	1.1–6.0	0.032	5.21	1.5–18.4	0.010
Duration of stay in KTM <2 years	2.27	1.0–5.0	0.044	2.29	0.9–5.7	0.077	1.32	0.6–2.8	0.475			
Local flooding	1.56	0.6–4.0	0.350				0.91	0.3–2.6	0.858			
Local unsanitary conditions^a^	0.70	0.3–1.5	0.361				0.97	0.4–2.1	0.942			
Use of stone spout water	1.36	0.4–4.3	0.592				3.70	1.2–11.2	0.020	4.17	0.9–20.5	0.078
Water stored after collection	1.23	0.5–3.0	0.636				3.09	1.2–8.1	0.022	2.56	0.8–8.6	0.129
Metal covering of water storage^b^	0.14	0.1–0.4	<0.001	0.19	0.1–0.7	0.014	0.22	0.1–0.6	0.003			
Latrine type												
Community	1.00	-					1.00	-		1.00	-	
Household	6.10	1.7–22.1	0.006	4.92	1.2–19.5	0.024	8.52	1.8–40.1	0.007	7.26	1.4–37.2	0.017
Eaten street food<2weeks	2.74	1.3–5.9	0.010	2.95	1.1–7.8	0.028	2.01	1.0–4.2	0.061			
Dairy consumption	1.08	0.2–6.7	0.938				1.41	0.4–5.3	0.609			
Pani puri consumption	0.30	0.1–0.8	0.021				0.56	0.2–1.4	0.217			

a : garbage or defecation visible in neighborhood;

b : inclusion of metal storage cover in the multivariate model for *S.* Typhi led to a model that did not converge.

### Specific risks for *S.* Typhi infection

Many of the important protective and risk factors for *S.* Typhi infection only were again similar to the overall enteric fever analysis, however some *S.* Typhi-specific exposures emerged. Firstly, the use of stone spout water and household water storage were strong risk factors for *S.* Typhi infection in the univariate analysis, although the use of stone spout water was only mildly significant in the multivariate model (AOR: 4.17, 95%CI: 0.9–20.5, p = 0.078). Water storage was a risk but was not significantly associated with *S.* Typhi infection in the multivariate model (AOR: 2.56, 95%CI: 0.8–8.6, p = 0.129). Living in a house with more than 12 people was also a risk that did not remain strongly significant in the multivariate model (AOR: 2.11, 95%CI: 0.8–5.5, p = 0.127), although reporting a monthly income of less than $125 was strongly and independently associated with *S.* Typhi infection (AOR: 5.21, 95%CI: 1.5–18.4, p = 0.010). Awareness of enteric fever was strongly protective, specifically against infection with *S.* Typhi (AOR: 0.28, 95%CI: 0.1–0.8, p = 0.014).

### *S.* Typhi and *S.* Paratyphi A serology

**Figure 2:**
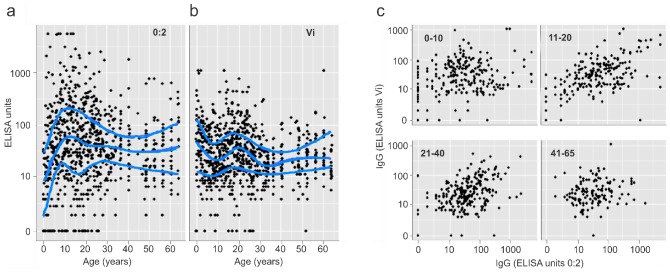
IgG serology against Vi-antigen (*S.* Typhi) and 0:2-antigen (*S.* Paratyphi A) in an age-stratified cross-section of the population of Kathmandu. a) Scatter plots showing antibody (IgG) levels against 0:2-antigen (left) and Vi-antigen in an age stratified population of Kathmandu, Nepal. Smoothed lines correspond to age-dependent median and quartiles which were estimated based on quantile regression with age included as a natural cubic spline function with 5 degrees of freedom. b) Age stratified scatter plots scatterplots (clockwise, 0–10 years, 11–20 years, 21–40 years and ≥40 years) of IgG 0:2 (x axis) and IgG Vi (y axis).

To assess exposure to *S.* Typhi and *S.* Paratyphi A in the local population, we measured IgG against Vi antigen (*S.* Typhi) and O:2 antigen (*S.* Paratyphi A) in 795 age-stratified (0–65 years) serum samples derived from the same population as our case/control enrollees (Figure 2). The resulting data demonstrated a consistently high level of IgG against Vi and O:2 in all age groups. IgG against *S.* Typhi was highest at birth and then declined with a secondary peak at the age of 17–18 years. Antibody against *S.* Paratyphi A was lowest at birth and peaked at the age of 11–12 years and subsequently declined; yet persisted into old age. There was a weak correlation between levels of IgG to Vi and to O:2 (Spearman's ρ: 0.30, *P*<0. 001), this was most apparent within the group aged 11–20 years (Spearman's ρ: 0.50, *P*<0.001) (Figure 2).

## Discussion

The aim of this study was to elucidate risk factors for, and protective behavior against, *S.* Typhi and *S.* Paratyphi A infection in Kathmandu. These analyses were performed in order to more clearly define the epidemiology of enteric fever for developing appropriately targeted control measures. We identified several risk factors and protective variables that could be targets for future intervention studies; in addition, our data show some differences in epidemiology between the two enteric fever serovars and highlight continued exposure and enteric fever risk as a function of daily life in this location.

Compared to *S.* Paratyphi A, enteric fever due to *S.* Typhi is historically thought to be more common, have a more severe clinical course, and result in more frequent and severe sequelae [27]. However, recent studies have suggested that infections caused by *S.* Paratyphi A are now more prevalent in areas endemic for enteric fever, and that infections caused by *S.* Typhi and *S.* Paratyphi A are clinically indistinguishable [8], [19], [28]–[33]. Our data in the present study support this trend. Whether this increase in *S.* Paratyphi A is a consequence of the decline of enteric fever due to *S.* Typhi or due to an absolute increase in the incidence of *S.* Paratyphi A is still not clear [19], [34], [35]. The relative increase in *S.* Paratyphi A has important implications for public health efforts to control the burden of disease. Particularly, the oral Ty21a and the parenteral Vi typhoid vaccines offer limited or no protection, respectively, against *S.* Paratyphi A [29], [36]. From our serological data, we suggest that vaccination with the currently available vaccines in many age groups may not dramatically impact the rates of disease in this population due to sustained exposure and high levels of pre-existing antibody. The dawn of Vi and O:2 conjugate vaccines may substantially reduce the burden of disease, but selecting the pivotal target population is paramount for the success of these vaccines.

When examining all enteric fever cases and matched controls, several factors that were protective against infection emerged. Notable protective variable included, an awareness of enteric fever, and the use of a metal cover on household water storage containers. The strong protective effect of a metal covering for water storage (as opposed to plastic) is likely explained by uncollected information associated with water treatment practices. We understand that households that filter their water prior to drinking are likely to have more permanent water storage containers with a metal covering; therefore it is likely that use of a ceramic filter explains the protective association of a metal covering on water storage units. We identified the use of a household latrine, apposed to a community latrine, as a significant risk for enteric fever in this analysis. Proximity of a household latrine to the kitchen or sleeping area of a family may explain this risk factor. In times of water shortage people are not be as likely to flush after each use, thereby exposing residents to contamination. The community latrines, however, are located at considerable distances from the living area of families in this setting and thus present a lesser risk of household fecal contamination. Further investigation into household toilet usage and behavior is warranted. Additionally, storage of water in houses with a large number of residents was found to be a risk for infection. Contamination of water stored in a household is common in regions where municipal water supply is unsafe [37]. Large numbers of people using one supply of stored water increases the opportunity for contamination due to increased contact with hands and utensils.

It has been suggested that *S.* Paratyphi A and *S.* Typhi may follow different transmission routes and the former requires a higher infectious dose for clinical disease [19]. Our exposure analysis supports this notion and suggests that while the two serovars share some risk factors and protective effects, the two organisms may have some independent epidemiology. In comparing various exposures between those with *S.* Typhi and *S.* Paratyphi A, *S.* Typhi cases were more likely to be associated with a poor-quality water source than the *S.* Paratyphi A cases. Water contamination with *S.* Typhi has been demonstrated previously in Nepal [16], [21]. The traditional stone spouts found throughout Kathmandu are supplied by a system of waterways with high risk of sewage contamination due to poor maintenance. High concentrations of *S.* Typhi and *S.* Paratyphi A DNA, in addition to fecal coliforms, have been reported from water samples from these stone spouts [10]. The fact that a majority of water-related and neighborhood condition exposures did not remain significantly associated with *S.* Typhi infection in the analysis is likely a result of our study design as both cases and controls within a particular neighborhood were likely to report using the same water sources. Finally, an additional risk factor specific for *S.* Typhi included a household income <$125/month; poor socioeconomic status is a well-known risk factor for infection with *S.* Typhi [13], [19].

For *S.* Paratyphi A induced enteric fever, two factors were found to be associated with risk of infection: residing in Kathmandu for less than years and eating street food in the two weeks preceding illness. Migration to Kathmandu from surrounding rural areas in search of economic prosperity is common and thus recent arrival into the city may correlate with immunologic naiveté. As *S.* Paratyphi A is an emerging pathogen in this setting [28], individuals from rural areas may not be exposed to the bacteria until arriving in Kathmandu. As *S.* Typhi has traditionally been the dominant serovar in this region, it is possible that transmission occurs throughout urban and rural Nepal [38]. Additionally, eating street food has been implicated in a previous study from Indonesia as a risk factor for infection with *S.* Paratyphi A as *S.* Paratyphi A reaches the required threshold to cause disease within certain food products [19]. As there is no hygiene legislation for street vendors in Kathmandu, such conditions and lengthy incubation periods are feasible.

There are some limitations with this study. Firstly, matching controls for ward of residence influenced the analysis, as the ward of residence is likely to be a correlate of various exposures. This altered the interpretation of the results, as many of the case/control pairs were concordant with regard to exposures, and did not contribute to the overall estimate of effect. Additionally, as shown by the serological data, identifying an appropriate group of controls in an endemic area poses a significant challenge. Given these challenges, we were still able to identify risks for enteric fever within this population that may allow for targeted interventions for the reduction of transmission. Such information is valuable as informed control and prevention strategies could provide a palatable and feasible method of reducing overall disease burden in this area of high endemicity.

 Historical surveillance data suggest that enteric fever rates decrease in parallel with the introduction of treatment of water supplies, and the exclusion of human feces from food production [39], [40]. Improvements in the infrastructure of the municipal water delivery system, in addition to the provision of a combined vaccine against both *S.* Typhi and *S.* Paratyphi A, would be optimum for eliminating enteric fever in Kathmandu. In the short to mid-term absence of these interventions, we advocate safer household water supplies through the use of small water filtration and storage systems [41]. Additionally, our data suggest that improvements in the quality of street food, as well as promotion of enteric fever and toilet hygiene awareness through campaigns educating the population on risks, symptoms and preventive measures would have the largest impact on the burden of enteric fever in Kathamandu.

## Supporting Information

Checklist S1 **STROBE checklist.** A complete case control STROBE checklist.
(DOC)

Table S1 **Case-control questionnaire.** A copy of the questionnaire administered to 103 cases and 294 controls in Patan Hospital and in the community, respectively, in a matched case control investigation conducted in Kathmandu, Nepal in 2011. There are a total of 131 questions detailing identification and demographic data, information on enteric fever exposures and past clinical history.
(DOCX)
