# Supplementary material for: Differential Epidemiology of Salmonella Typhi and Paratyphi A in Kathmandu, Nepal: A Matched Case Control Investigation in a Highly Endemic Enteric Fever Setting
Source: PLoS Negl Trop Dis. 2013 Aug 22;7(8):e2391. doi: 10.1371/journal.pntd.0002391 (PMC3749961; doi:10.1371/journal.pntd.0002391)
Supplement: Table S1 — Case-control questionnaire. A copy of the questionnaire administered to 103 cases and 294 controls in Patan Hospital and in the community, respectively, in a matched case control investigation conducted in Kathmandu, Nepal in 2011. There are a total of 131 questions detailing identification and demographic data, information on enteric fever exposures and past clinical history. (DOCX) [file pntd.0002391.s002.docx]

**Case control study**

**Patan Hospital-Oxford University Clinical Research Unit, Nepal**

**Section 1- Registration**

| 1.1 | Study Registration number |  |  |
| --- | --- | --- | --- |
| 1.2 | Date of interview (Western)  DD/MM/YYYY |  |  |
| 1.3 | Interview done with | Respondent  Parent of respondent  Relative of respondent  Friend |  |

**Section 2 – Identification and Demographic data**

For Cases and Controls

| 2.1 | Participant status | Case  Concurrent case  Control  Concurrent control |  |
| --- | --- | --- | --- |
| 2.2 | If control or concurrent case, write the corresponding case’s study ID number |  |  |
| 2.3 | Name |  |  |
| 2.4 | Sex | Male  Female |  |
| 2.5.1 | Date of Birth (Western)  (DD/MM/YYYY) |  |  |
| 2.5.2 | Date of Birth (Nepali; if Western unknown)(DD/MM/YYYY) |  |  |
| 2.6 | Religious group | Hindu  Buddhism  Muslim  Christianity  Others ____________ |  |

| 2.7.1a | **Permanent** Address: Area |  |  |
| --- | --- | --- | --- |
| 2.7.1b | Address: Ward No. |  |  |
| 2.7.1c | Address: Town/Village |  |  |
| 2.7.1d | Address: District |  |  |
| 2.7.2a | **Temporary** Address: Area |  |  |
| 2.7.2b | Address: Ward No. |  |  |
| 2.7.2c | Address: Town/Village |  |  |
| 2.7.2d | Address: District |  |  |
| 2.8.1 | Telephone number: Home |  |  |
| 2.8.2 | Telephone number: Cell |  |  |
| 2.9 | GPS locations |  |  |
| 2.10 | Which address is the current address? | Temporary  Permanent |  |
| 2.11 | How long have you been living at the current address? | Less than 1 month  Less than 2 months  Less than 6 months  Less than 1 year  Less than 2 years  More than 2 years |  |
| 2.12 | Do you go back to your hometown from time to time? | Yes  No |  |
| 2.13 | When was the last time you went back to your hometown? | _____ months ago |  |
| 2.14 | How long did you stay in your hometown? | _____days/ months/ years |  |
| 2.15 | Have you spent more than one month out of the last year living outside Kathmandu? | Yes  No |  |
| 2.16 | If yes, for how long? | _____ days/ months/ years |  |
| 2.17 | Why are you in Kathmandu? | Study  Work  Business  Personal |  |
| 2.18 | Daily Activity | Student  Work  Housewife  Unemployed |  |
| 2.19 | Education level of the respondent | Illiterate  Never attended primary school but can read and write  Primary school not finished  Primary school finished  Junior high school  Senior high school  Higher education |  |
| 2.20 | Type of profession of the respondent | Student  Worker  Civil service  Private company  Entrepreneur  Free professions (Doctor, lawyer, Consultant)  Housewife  Unemployed |  |

| 2.21 | Field of profession of the respondent | Agriculture  Industry and manual labour  Public utility (Electricity, gas and water)  Construction  Trade and commerce (shopkeeper)  Transport, Distribution and communication  Commerce, Finance, Insurance, Leasing  Civil Service  Armed forces  Domestic work  Food preparation: cook, street vendor, restaurant keeper, Street vendor  Manufacturing (carpet factory, metal works)  Other |  |
| --- | --- | --- | --- |
| 2.22 | Location of work/ school of respondent |  |  |
| 2.22.1a | Work Address: Area |  |  |
| 2.22.1b | Ward No. |  |  |
| 2.22.1c | Town/Village |  |  |
| 2.22.1d | District |  |  |
| 2.23 | How long have you been studying/ working here? | 0-6 months  Less than one year  Less than 2 years  More than 2 years |  |

**For Cases**

| 2.24 | Hospital number |  |  |
| --- | --- | --- | --- |
| 2.25 | Laboratory number |  |  |
| 2.26 | Microbiology result | *S.* Typhi  *S*. Paratyphi A  *S.* Paratyphi B  *S.* Paratyphi C  Other Salmonellae  No growth  Other |  |

**Section 3 – Questionnaire**

| 3.1 | Did you / the child have fever in the past three days? | Yes  No  Not sure |  |
| --- | --- | --- | --- |
| 3.2 | If yes, what was the pattern of fever | Continuous  Intermittent  Remittent |  |
| 3.3 | For how long have you/ the child been suffering from the fever? | Days |  |
| 3.4 | Did you/ the child have any of the following symptoms in the past week? | Fever at night Y/N  Progressive fever Y/N  Fever longer  than 2 weeks Y/N  Anorexia Y/N  Nausea Y/N  Abdominal pain Y/N  Diarrhea Y/N  Constipation Y/N  Rectal blood loss Y/N  Cough Y/N  Headache Y/N  Impaired  consciousness Y/N  Petechiae and  rose spots: skin spots Y/N  Dark colour of urine Y/N  Rhinorrhea Y/N |  |
| 3.5 | Do you/ the child have any existing chronic illness? | Yes  No  Not sure |  |
| 3.6 | Do you know what typhoid fever is? | Yes  No  Not sure |  |
| 3.7 | Can you name 3 of the most prominent symptoms in order of severity? | Yes  No  Choices ________ |  |
| 3.8 | Do you know where you catch it from? | Ill people Y/N/DK  Food Y/N/DK  Water Y/N/DK  Air Y/N/DK  Body fluids Y/N/DK  Mosquito Y/N/DK  Fly Y/N/DK  Human faeces Y/N/DK  Coughing Y/N/DK  Hands Y/N/DK  Animal faeces Y/N/DK  Rats Y/N/DK  Cockroach Y/N/DK  Unsafe sex Y/N/DK |  |
| 3.9 | Did you/ child have typhoid fever before? | Yes  No  Not sure |  |
| 3.10 | If yes, was it culture confirmed? | Yes  No  Not sure |  |
| 3.11 | Did anybody in the household have typhoid fever in the past 8 weeks? | Yes  No  Not sure |  |
| 3.12 | Did you/ the child have contact with typhoid patient in past 8 weeks? | Yes  No  Not sure |  |
| 3.13 | Did you/ the child visit a health care provider in the past one week? | Yes  No  Not sure |  |
| 3.14 | Did you/ the child receive any medication in the past week for this episode of fever? | Yes  No  Not sure |  |
| 3.15 | What type of medicine did you/ the child take? | Don’t know  Antibiotic  Antipyretic  Others……………….. |  |
| 3.16 | What antibiotics have you/ the child received for this episode of fever? | Don’t know  Chloramphenicol  Amoxicillin  Cefixime  Ciprofloxacin  Ceftriaxone  TMP-SMX  Azithromycin  Ofloxacin  Other _________________ |  |
| 3.17 | For how many days did you/ the child take the medication? | Days |  |
| **Socio economic conditions** | | | |
| 3.18 | With whom do you live at the moment? | Together with family  With friends  Alone  Orphanage  Hostel  Others ……………… |  |
| 3.19 | Who is the owner of the house | Respondent  Family  Rent  Others ……………….. |  |
| 3.20 | What is the rent per month | Rupees …………. |  |
| 3.21 | Is your house affected by rainfall in the monsoons? | Yes  No  Only some years with heavy rainfall |  |
| 3.22 | How is your house affected during the rainfall? | Mild flooding  Complete flooding of the ground floor  Others. Define |  |
| 3.23 | Is your neighbourhood affected by rainfall in the monsoons? | Yes  No  Only some years with heavy rainfall |  |
| 3.24 | How is your neighbourhood affected during the rainfall? | Mild flooding  Severe flooding  Others. Define |  |
| 3.25 | Do you notice overflowing water pipes or sewage pipes in your neighbourhood during the monsoons? | Yes  No  Have not noticed |  |
| 3.26 | Is there an open garbage disposal point next to/ near your house? | Yes  No  Have not noticed |  |
| 3.27 | Do people or animals defecate on the streets in your neighbourhood? | Yes  No  Have not noticed |  |
| 3.28 | How many people live in your household? |  |  |
| 3.29 | How many children are in your household? |  |  |
| 3.30 | How many people sleep in one room? |  |  |
| 3.31 | Do you have a radio? | Yes/No |  |
| 3.32 | Do you have a TV? | Yes/No |  |
| 3.33 | Do you have a bicycle? | Yes/ No |  |
| 3.34 | Do you have a motorbike? | Yes/No |  |
| 3.35 | Do you have a car? | Yes/No |  |
| 3.36 | Do you have a fridge? | Yes/No |  |
| 3.37 | Do you have a telephone? | Yes/No |  |
| 3.48 | Do you have a mobile phone? | Yes/No |  |
| 3.39 | How many family members are earning? (Any contribution counts) |  |  |
| 3.40 | What is the total household monthly income? | Less than 3000 NRs  Less than 5000 NRs  Less than 10000 NRS  Less than 15000 NRs  Less than 20000 NRs  More than 20000 NRs |  |
| 3.41 | How many people depend on the income? |  |  |
| 3.42 | What is the average monthly expenditure of the household? | Less than 3000 NRs  Less than 5000 NRs  Less than 10000 NRS  Less than 15000 NRs  Less than 20000 NRs  More than 20000 NRs |  |
| **Water source** | | | |
| 3.43 | What is the main source of drinking water at home? | Jar (what is a jar?)  Mineral (bottled) water  Stone Spout  Well  Hand pump  Piped water supply  Private company water  River water  Other. Define_______ |  |
| 3.44 | Does your house have municipal supplied water? | Yes  No |  |
| 3.45 | If yes, how many days a week do you get municipal supply? | Once  Twice  Thrice  Four times  Don’t know |  |
| 3.46 | Do you store the municipal supplied water? | Yes  No |  |
| 3.47 | If no municipal supply/ or if municipal supply is not enough, where do you get your water from? | Stone spout  Well  Private water company  River  Rainfall |  |
| 3.48 | Is water stored after collection from the source? | Yes/No |  |
| 3.49 | Do you have a major storage area? | Yes/No |  |
| 3.50 | If yes, what do you store it in | Plastic storage tanks  Concrete storage tanks  Metal storage tanks  Others………………... |  |
| 3.51 | Just before use, what do you store it in? | Jerry can  Gagri  Surai  Bottles  Jars  Buckets  Kitchen utensils  Others………………... |  |
| 3.52 | What is the mouth of the container like? | Narrow  Wide |  |
| 3.53 | Is the stored water container covered? | Yes  No |  |
| 3.54 | What is the material of the storage container? | Clay  Concrete  Plastic  Aluminum  Copper  Brass  Others………………... |  |
| 3.55 | Do you know the location of your nearest water spout (name) | Yes  No  Name………………… |  |
| 3.56 | Are major water storage areas cleaned? | Yes  No  Don’t know |  |
| 3.57 | If yes, how often? | Monthly  Twice a year  Annually  Never |  |
| 3.58 | How do you treat the drinking water at home? | Do not treat  Boil  Filter  Chlorinate  SODIS  Others……………… |  |
| 3.59 | What is the source of water for household chores (washing dishes, clothes etc)? | Collected rain water  Stone Spout  Well  Hand pump  Piped water supply  Private water company  Others……………….. |  |
| 3.60 | What is the source of water for washing your hands before eating? | Collected rain water  Stone Spout  Well  Hand pump  Piped water supply  Water bought from private company |  |
| 3.61 | What is the source of water for washing your hands after defecation? | Collected rain water  Stone Spout  Well  Hand pump  Piped water supply  Water bought from private company |  |
| 3.62 | What water do you use for bathing? | Collected rain water  Stone Spout  Well  Hand pump  Piped water supply  Water bought from private company |  |
| 3.63 | What water do you drink at work/ school? | Jar  Mineral (bottled) water  Stone Spout  Well  Hand pump  Piped water supply  Private company water  River water  Other. Define _______ |  |
| **Personal Hygiene** | | | |
| 3.64 | Does water shortage affect your daily activities (bathing, washing clothes etc) | Yes/No |  |
| 3.65 | Do you wash your hands before eating? | Always  Often  Sometimes  Never |  |
| 3.66 | If yes, how do you wash your hands? | Water and soap  Water only  Ash |  |
| 3.67 | Do you wipe your hands after washing? | Yes/No |  |
| 3.68 | If yes, what do you wipe it on? | Towel  Clothes  Other |  |
| 3.69 | What is the type of latrine used by the household? | No toilet present  Household latrine  Community latrine  River  Gutter  Field |  |
| 3.70 | If a latrine is used, where is it located? | Indoor  Outdoor |  |
| 3.71 | If a latrine is used, how many people share it? |  |  |
| 3.72 | Does it have water flushing system? | Yes  No |  |
| 3.73 | Do you wash hands after defecation? | Yes  No |  |
| 3.74 | What do you wash your hands with? | Soap  Only water  Ash  Others………………... |  |
| **Eating habits** | | | |
| 3.75 | Do you have a kitchen? | Yes  No |  |
| 3.76 | How many people share the kitchen? |  |  |
| 3.77 | Do you eat with your hands? | Always  Often (more than half the time)  Seldom  Never |  |
| 3.78 | Who prepares food in the household? | No one, eat out  Respondent  Family member  Other household member  Domestic help |  |
| 3.79 | Do you wash your hands before preparing food? | Always  Often  Never |  |
| 3.80 | What do you wash your hands with? | Soap  Water  Do not wash |  |
| 3.81 | What is the source of water for washing your hands before food preparation? | Municipal supply tap water  Private water company provided tap water  Protected well  Uncovered well  Stone spout  River water  Collected rain water  Stored water  Others………………... |  |
| 3.82 | What is the source of water for washing your vegetables, fruits and kitchen utensils? | Municipal supply tap water  Private water company provided tap water  Protected well  Uncovered well  Stone spout  River water  Collected rain water  Stored water  Others………………... |  |
| 3.83 | How often do you eat cooked food sold by street vendors? | Never  ____ times/day  ___ times/week  ___times/month  Not sure |  |
| 3.84 | How often do you eat sliced fruit sold by street vendors? | Never  ____ times/day  ___ times/week  ___times/month  Not sure |  |
| 3.85 | How often do you eat ice cream sold by street vendors? | Never  ____ times/day  ___ times/week  ___times/month  Not sure |  |
| 3.86 | How often do you eat milk or dairy products from street vendors? | Never  ____ times/day  ___ times/week  ___times/month  Not sure |  |
| 3.87 | Where do you get your milk or dairy products from? | DDC  ND  Grocery shop  Private farmer  Not sure |  |
| 3.88 | How often do you eat pani puri, chat sold by street vendors? | Never  ____ times/day  ___ times/week  ___times/month  Not sure |  |
| 3.89 | Did you recently (last two weeks) eat food sold by street vendors? | Yes  No  Not Sure |  |
| 3.90 | Did you recently (last two weeks) eat at a restaurant? | Yes  No  Not sure |  |
| 3.91 | Do you buy food at street vendors to eat at home? | Yes  No |  |
| 3.92 | If yes, how often? | Every day  3-4 times weekly  Once a week  Less than once a week |  |
| 3.93 | Do you use ice cubes in your drinks? | Yes  No |  |
| 3.94 | Where do you get your ice cubes from? | From own fridge  From the restaurant  From the ice vendor |  |
| 3.95 | How do you make your ice cubes? | Mineral water  Boiled water  Untreated tap water  Untreated other water |  |

**Section 4 – Vaccination History**

| 4.1 | Have you ever received vaccines for typhoid? | Yes  No  Not sure |  |
| --- | --- | --- | --- |
| 4.2 | If yes, where did you/ the child receive the vaccine? | School  Hospital _______  Clinic ________  Other ­­­­_________ |  |
| 4.3 | If yes, what was the date in which you/ the child received the vaccine (Western date dd/mm/yyyy) |  |  |
| 4.4 | Did anyone else in the household receive typhoid vaccine? | Yes  No |  |
| 4.5 | If yes, how many people received the vaccine? |  |  |

**Section 5 – Administration**

| 5.1 | Was the interview complete? | Yes  No |  |
| --- | --- | --- | --- |
| 5.2 | If no, what was / were the reason(s)? | Refusal  Time constraints  Comprehension  Lack of interests  Other ………………… |  |
